# From Fabrication to Failure—Aqueous Processing, Electrolyte Tuning, and Degradation Mechanism Elucidation in Poly(3‐Vinyl‐*N*‐Methylphenoxazine) Positive Electrodes

**DOI:** 10.1002/cssc.202500753

**Published:** 2025-08-10

**Authors:** Sathiya Priya Panjalingam, Somayeh Ahadi, Markus Börner, Birgit Esser, Martin Winter, Peter Bieker

**Affiliations:** ^1^ MEET Battery Research Center Institute of Physical Chemistry University of Münster Corrensstr. 46 48149 Münster Germany; ^2^ International Graduate School for Battery Chemistry Characterization Analysis Recycling and Application (BACCARA) Corrensstr. 40 48149 Münster Germany; ^3^ Institute of Organic Chemistry II and Advanced Materials Ulm University Albert‐Einstein‐Allee 11 89081 Ulm Germany; ^4^ Helmholtz Institute Münster (HI MS) IMD‐4 Forschungszentrum Jülich GmbH Corrensstr. 46 48149 Münster Germany

**Keywords:** aqueous binders, electrolyte, lithium organic battery, poly(vinylphenoxazine)

## Abstract

Organic electrode materials are considered the next generation of battery electrode materials due to their environmental friendliness, low toxicity, and competitive specific capacities. Herein, a systematic study of the processing of the redox polymer poly(3‐vinyl‐*N*‐methylphenoxazine) (PVMPO) using water‐processable binders, achieving comparable performance comparable to that of PVdF‐based electrode, is reported. To address the dissolution of the oxidized polymer into the electrolyte, two different electrolytes are investigated to maximize specific capacity. The use of 1.0 m LiPF_6_ in 3:7 EC:EMC electrolyte inhibits the dissolution of the oxidized polymer, as confirmed by UV–Visible spectroscopy, *ex situ* scanning electron microscopy (SEM) analysis after the 1st charge and 1st discharge, and cyclic voltammetry measurements. The inhibition results in 79% capacity retention at the end of 500 cycles. Additionally, the polymer demonstrates notable rate capability even at 100C, with capacity retention of 82% after 10,000 cycles. This performance is attributed to the faster kinetics resulting from the planar geometry of phenoxazine units in PVMPO. However, notable capacity fading is observed after 200 cycles when cycling at 1C rate, found to be due to polymer decomposition. This study opens up a new avenue for exploring aqueous processing of redox polymers and potential reasons for capacity fading.

## Graphical abstract

Organic electrode materials are considered the next generation of battery electrode materials due to their environmental friendliness, low toxicity and competitive specific capacities. Herein, we report on a systematic study of processing of the redox polymer poly(3‐vinyl‐N‐methylphenoxazine) (PVMPO) using water processable binders, achieving comparable performance to that of PVdF‐based electrode.

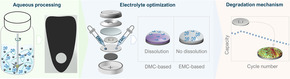

## 1 Introduction

The 21st century calls for ‘efficient’ and ‘sustainable’ energy storage. The ever‐increasing energy demand accelerates the need for high‐energy‐density storage devices.^[^ ^1^ ^]^ The Nobel Laureate Richard Smalley's 'Terawatt Challenge’ places energy at the forefront and emphasizes the need for sustainable energy production and efficient storage.^[^ ^2^, ^3^ ^]^ While lithium ion batteries (LIBs) play a major role in the electrification of transportation, grid storage, and portable electronics, the 2021 materials report predicts that the cost of LIBs will mainly depend on cathode materials.^[^ ^1^ ^]^ Currently, cobalt (Co) and nickel (Ni) are widely used in high‐energy density LIBs; however, by 2045, their demand is projected to outpace supply, leading to price volatility.^[^ ^1^ ^]^ In this regard, organic materials primarily composed of carbon, oxygen, nitrogen, sulfur, and hydrogen have gained attention.^[^ ^4^, ^5^, ^6^, ^7^, ^8^, ^9^ ^]^

Phenoxazine is a class of materials reported as high‐voltage *p*‐type positive electrode materials, known for fast charge transfer due to their planar molecular structure.^[^ ^10^ ^]^ Derivatives of phenoxazine have been explored in various electrochemical applications, including as redox shuttle additives and mediators for redox targeting.^[^ ^11^, ^12^, ^13^, ^14^, ^15^ ^]^ Zhang et al. have demonstrated phenoxazine‐based positive electrode polymer achieving 77% of the theoretical specific capacity (89.5 mAh g^−1^) after 500 cycles.^[^ ^12^ ^]^ Ning et al. also reported phenoxazine as a positive electrode material for aqueous zinc‐ion batteries.^[^ ^16^ ^]^

A previous study reported poly(3‐vinyl‐*N*‐methylphenoxazine) (PVMPO) as a promising *p*‐type material for lithium–organic batteries, showing good rate capability, cycling stability, and reasonable theoretical capacity of 120 mAh g^−1^ considering a one‐electron redox and a discharge potential of 3.52 V versus Li|Li^+^. Cross‐linking was found to inhibit solubility in the electrolyte.^[^ ^17^ ^]^ However, the electrode processing involves the hazardous poly(vinylidene difluoride) (PVdF) binder and *N*‐methyl‐2‐pyrrolidone (NMP) solvent /dispersant.^[^ ^17^, ^18^ ^]^

As an alternative, water‐based, fluorine‐free binders such as carboxymethyl cellulose (CMC) and styrene‐butadiene rubber (SBR) have emerged.^[^ ^18^ ^]^ CMC, derived from cellulose, is water‐soluble, known for its role as a thickening agent and dispersant/surfactant, and enhances electrode adhesion.^[^ ^18^, ^19^, ^20^ ^]^ SBR, an aromatic polymer produced by the copolymerization of styrene and butadiene, provides mechanical stability and heat resistance.^[^ ^18^, ^21^, ^22^ ^]^ CMC/SBR binder combination thus offers a sustainable processing route with high adhesion, cycling stability, strong dispersion, and better mechanical stability during volume expansion in the electrodes.^[^ ^18^, ^22^ ^]^ Inspired by the aqueous processing of state‐of‐the‐art negative electrodes, the aqueous processing of the inorganic positive electrodes gained much attention over the last decades.^[^ ^23^, ^24^, ^25^, ^26^ ^]^ Electrode fabrication of organic materials, however, has rarely explored aqueous binders.^[^ ^12^, ^17^, ^27^, ^28^, ^29^, ^30^, ^31^, ^32^, ^33^, ^34^ ^]^ Thus, employing PVdF and NMP in organic electrodes undermines their inherent environmental benefits. This study focuses on evaluating the performance of PVMPO with water‐soluble binders as sustainable alternatives.

Otteny et al.^[^ ^17^ ^]^ showed that PVMPO undergoes discharge similar to that of PVMPT.^[^ ^35^ ^]^ The polymer oxidizes to a di‐cationic state via a radical cation, which is soluble in an electrolyte. However, the di‐cationic state was not reversible, leading to semi‐oxidized deposition and capacity limitation.^[^ ^17^ ^]^ The solubility of the oxidized polymer in the electrolyte is a critical challenge. Reported mitigation strategies include cross‐linking,^[^ ^17^ ^]^ encapsulation with highly porous conductive additives,^[^ ^28^ ^]^ and optimizing the battery electrolyte.^[^ ^27^ ^]^ In order to get a cross‐linked polymer, a cross‐linker is necessary, which requires additional synthetic steps as reported by Otteny et al.^[^ ^17^ ^]^ Encapsulation also requires polymer solubility in the processing solvent; it is not applicable herein because the polymer does not dissolve in water. Thus, electrolyte optimization presents a practical solution.

Herein, the electrochemical performance of PVMPO with different aqueous binders was investigated, using an NMP‐based system as a reference. Rheology, adhesion, and conductivity measurements were compared across the binder systems. Electrochemical cycling at 1C for 1,000 cycles and electrolyte optimization were conducted to mitigate polymer dissolution and enhance capacity. The study also examines PVMPO's high‐rate capability and potential causes of capacity fading.

## 2 Experimental Section

### 2.1 Different Binder Compositions Used in this Study

This study investigates two primary binder systems (PVdF and CMC) and three binary binder systems (CMC combined with SBR). The binary binder system of CMC and SBR was prepared in three different ratios, as shown in **Table** **1**. The performance of the PVdF electrode system served as a reference for comparison with other aqueous systems. For aqueous binders, all binders were prepared as solutions to ensure homogeneous mixing with water.

**Table 1:** Weight percentage (composition) of the CMC and SBR binders used in this study.

Binder	CMC	CS55	CS73	CS82
CMC (%)	100	50	70	80
SBR (%)	–	50	30	20

### 2.2 Synthesis of PVMPO

PVMPO was synthesized as reported in ref. [17]. Powder X‐ray Diffraction, Fourier transform infrared spectroscopy, Raman spectroscopy, and SEM were used to characterize the as‐synthesized polymer, including phase identification, nature of bonds, and the morphology of the polymer.

### 2.3 Electrode Processing

Electrodes were prepared by mixing the as‐synthesized PVMPO powder, the commercially available conductive additive carbon black (Super C65, Imerys Graphite & Carbon), poly(vinylidene difluoride) (PVdF, Solef 5130, Solvay), sodium CMC (Na‐CMC, CRT 200 PPA12, Dowwolff Cellulosics, Germany), SBR (Synthomer, 50% SBR, 50% H_2_O). Electrode pastes for non‐aqueous systems, i.e., PVdF‐based electrode, were prepared by mixing PVMPO, carbon black, and PVdF by adding the required amount of NMP (battery grade, Sigma Aldrich) in the Thinky mixer (Arm–310) for 1 h at 1700 rpm in the weight ratio of 50:45:5, respectively. However, the same technique could not be followed for the aqueous electrode pastes as the material was not properly dispersed using only the Thinky mixer. So, for aqueous systems, initially, the solid components were hand‐mixed using a mortar and pestle for 30 min followed by the addition of the binder solution and the required amount of water in the same weight ratio (50:45:5). Once the required electrode paste consistency was achieved, the mixture was mixed in Thinky mixer for 1 h at 1700 rpm. The electrode pastes obtained were cast on 5 wt% (KOH) potassium hydroxide‐etched aluminum foil (Speira, thickness: 20 μm) by doctor blade coating (Zehntner 4‐sided film applicator) (50 μm spacing). The resulting electrodes were dried in a hot air oven for 1 h (primary drying) and a vacuum drying cabinet at 70 °C overnight (secondary drying). Electrode discs of 12 mm diameter were prepared, pressed in a hydraulic press at 3 tons pressure for 20 s, and further dried at 80 °C (for non‐aqueous discs) and 120 °C (aqueous discs) under reduced pressure of 0.005 mbar for 12 h in a Büchi B‐585 glass oven. The resulting electrodes had dry film thicknesses of 10–13 μm (excluding the thickness of the current collector) and active mass loadings between 0.2–0.4 mg cm^−2^.

### 2.4 Cell Assembly and Electrochemical Measurements

The electrochemical performance of the PVMPO composite electrodes prepared by nonaqueous and aqueous methods was evaluated using a three‐electrode Swagelok T‐cell setup. The half‐cells employed lithium metal as the counter (12 mm disc) and reference electrode (5 mm disc), with PVMPO composite electrodes serving as the working electrode. The electrolyte used for the binder study was 1.0 m (LiPF_6_) lithium hexafluorophosphate in 1:1 (EC) ethylene carbonate:(DMC) dimethyl carbonate v/v (Sigma‐Aldrich), while for the electrolyte comparison study, in‐house prepared 1.0 m LiPF_6_ in 1:1 EC:DMC and 1.0 m LiPF_6_ in 3:7 EC:(EMC) ethyl methyl carbonate by weight were used. The electrolytes were prepared in an MBraun glovebox under a nitrogen atmosphere, with water and oxygen levels maintained at 0.01 ppm. Six layers of Freudenberg 2190 non‐woven polypropylene separator soaked with 130 μL of electrolyte, were used for the counter electrode and the PVMPO composite electrode (13 mm separator), while 60 μL was used at the reference electrode (10 mm separator). The separators were punched and predried under reduced pressure (0.005 mbar) at 120 °C for 24 h in a Büchi B‐585 glass oven before cell assembly. The electrolytes were dried overnight over molecular sieves (activated at 300 °C for 24 h under reduced pressure) prior to cell assembly. The cells were assembled in a dry room with humidity below 20 ppm and allowed to rest for 12 h before cycling. Galvanostatic cycling investigations were performed using a MACCOR 4000 series battery tester at various C‐rates, as specified in the corresponding figure captions, operating within a voltage range of 3.0 to 4.0 V. Cyclic voltammetry (CV) was performed with Swagelok T‐cells using a Biologic VMP3 potentiostat (Science Instruments) at 20 °C in a Binder oven, within a voltage window of 3.0 V to 4.0 V. *In situ* potentiostatic electrochemical impedance spectroscopy (PEIS) measurements were taken after a relaxation period of 12 h and between cycles, cycled at a 1C rate in the half‐cells. The measurements were conducted across a frequency range of 1 MHz to 20 mHz with an amplitude of 10 mV, using the Biologic VMP3 potentiostat (Science Instruments) at 20 °C inside a Binder oven. The obtained EIS data were analyzed by fitting to an equivalent circuit model using RelaxIS 3 software (RHD instruments).

## 3 Physical Characterization

### 3.1 X‐Ray Diffraction (XRD)

The powder XRD pattern of the as‐synthesized PVMPO polymer was acquired by using a Bruker D8 Advance with Cu kα radiation (1.54 Å) using a Bragg–Brentano geometry radiation between the 2θ range of 5°–90° with the recording time of 2 s per step with a step size of 0.02° s^−1^ at 40 kV and 20 mA. A 0.5 m divergence slit was used for the measurements.

### 3.2 Fourier Transform‐Infrared (FT‐IR) Spectroscopy

The FT‐IR spectrum of PVMPO was measured with a Bruker Vertex 70 in the mid IR region between 400–4000 cm^−1^ with single‐reflection‐attenuated total reflectance (ATR) (diamond ATR Unit) and KBr beam splitter. The chamber was flushed with nitrogen (N_2_) gas for one hour prior to measurement, and the spectrum was recorded with a constant N_2_ flow.

The FT‐IR spectra of bare lithium, the EMC‐based electrolyte, and cycled lithium were recorded using a Bruker ALPHA II spectrometer in an argon atmosphere (glove box). The samples were positioned onto the ATR crystal, with a wavenumber range of 400–4000 cm^−1^.

### 3.3 Raman Spectroscopy

The Raman spectrum of PVMPO was obtained using a confocal microscope (Horiba Scientific, LabRAM HR Evolution) equipped with an air‐cooled CCD detector and a 600 g mm^−1^ grating. A red laser (633 nm) with a power output of 10.5 mW at the objective was used for excitation, with the laser power reduced to 1.05 mW using a 10% filter. The laser was focused with a 50 × objective lens (Carl Zeiss Microscopy, 9.2 mm working distance, numerical aperture 0.5). Raman spectra were collected through three integrations of 35 s each. LabSpec 6.7.1.10 software (Horiba Scientific) was used for data acquisition, analysis, and control of the spectrometer. Before each measurement, the system was calibrated to the crystalline silicon peak at 520.7 cm^−1^.

### 3.4 Scanning Electron Microscopy (SEM)

SEM micrographs of the PVMPO polymer powder and PVMPO composite electrodes (pristine as well as *ex situ*) were measured on a Carl Zeiss AURIGA Crossbeam Workstation at an accelerating voltage of 3 kV with an in‐lens detector at a working distance of 4 mm. For *ex situ* measurements, the samples were transferred using a vacuum‐sealed sample holder to avoid exposure to air. For energy dispersive X‐Ray spectroscopy (EDS), the accelerating voltage used was 6 kV.

For lithium metal, SEM images were obtained on a Zeiss Crossbeam 550 electron microscope (Carl Zeiss Microscopy GmbH) at an accelerating voltage of 3 kV using an in‐lens detector at a working distance of 5 mm. To prevent air exposure, all Li‐metal samples were transferred using a vacuum‐sealed sample holder. For EDS, an Ultim Extreme detector was used to evaluate the elemental composition of the Lithium metal at an accelerating voltage of 5 kV.

### 3.5 Rheology

The rheological properties of the prepared electrode pastes were studied using a rheometer (Anton Paar MCR 102) with cone‐plate geometry (CP 150). The shear rate range was between 10 s^−1^ and 1000 s^−1^. 0.9 mL paste of all five different binders was transferred to the cone plate of the rheometer for the measurements.

### 3.6 Adhesion Strength

An adhesion tester (Z 2.5 by Zwick/Roell GmbH & Co. KG) was employed to assess the adhesive strength of electrodes through peel‐off tests. Steel sample holders (surface area: 6.45 cm^2^ per spot, with 3 spots per sample) were prepared using double‐sided adhesive tape to secure the samples. To ensure proper interaction between the sample and the holder, the upper sample holder was pressed onto the sample with a force of 2000 N for 60 s before pull‐off. The tests were performed at a pull‐off speed of 10 mm min^−1^ in the vertical direction.

### 3.7 Through‐Plane Conductivity (TPC) Measurements

The through‐plane resistance (TPR) was determined using the advanced two‐point method on a universal material testing machine (zwickiLine Z 2.5 with a 200 N Xforce load cell, Zwick/Roell GmbH & Co. KG). The dried electrode sheets after coating were placed between two copper stamps, and different pressures were applied. The resistance perpendicular to the electrode plane (TPR, R) was measured using an Ohmmeter (Resistomat 2316, Burster). The obtained resistance was used to calculate the TPC of the electrodes. The contact pressure was 77.5 kPa for the measurements, which is similar to the cell pressure values for high‐energy‐density applications.^[^ ^36^ ^]^

To determine the electronic conductivity of the electrodes, TPC measurements were conducted. The TPR was measured as a function of the applied pressure on the electrode sheets, and the corresponding TPC was calculated. Herein, TPR was measured by the two‐point current transit method at different pressures as proposed by Westphal et al.^[^ ^36^ ^]^ Thus, the TPC *σ* of the electrode sheets was calculated using the equation

$$\sigma =\frac{l}{R•A}$$

where *l* is the electrode thickness along with the current collector and *A* represents the contact area of the stamps (6.45 cm^2^), and *R* is the measured resistance.

### 3.8 UV–Vis Spectroscopy

UV–Vis spectroscopic measurements of the electrolyte were measured with a Shimadzu UV‐2450 spectrometer using a sealed quartz glass cuvette (115‐QS, Hellma Analytics) with a path length of 10 mm from 200 to 800 nm. The charged and discharged cells were disassembled in the glove box (O_2_ < 0.1 ppm; H_2_O < 0.1 ppm), and the separator was placed in a 1.5 mL Safe‐Lock Tube with a spacer (Eppendorf). The sealed lock tube was placed in the centrifuge (Galaxy SD Microcentrifuge, VWR International GmbH, Germany), and the electrolyte was extracted from the separator at 8500 rpm for 5 min. Then, 10 μl of the electrolyte extracted was diluted with 1000μl (1 mL) of acetonitrile (ACN) in the quartz vial for measurements.

## 4 Results and Discussion

### 4.1 Structural Characterization of the Synthesized Polymer PVMPO

**Figure 1:**
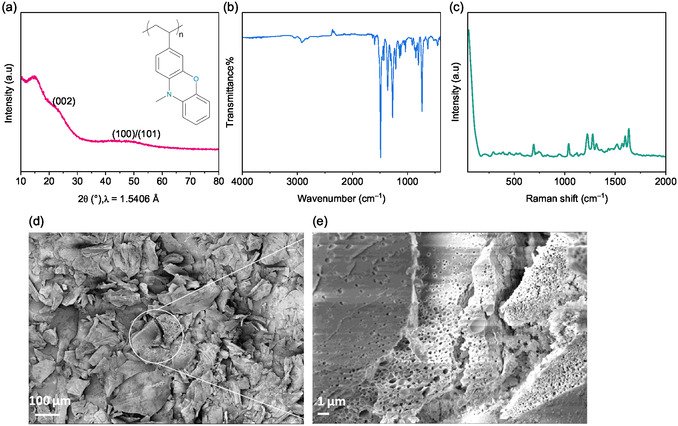
Physical characterization of PVMPO. a) XRD inset showing the chemical structure of PVMPO, b) FT‐IR spectrum, c) Raman spectrum, d,e) SEM images of the as‐synthesized PVMPO polymer at lower and higher magnifications, respectively.

**Figure** **1**a shows the XRD pattern of the as‐synthesized PVMPO, and the inset in Figure 1a shows the structure of PVMPO. It shows two broad reflections around 24° and 47° arising from (002) and (100)/(101) planes, which confirms the amorphous nature of the polymer. Amorphous systems possess short‐range order, which gives these materials several interesting and unique features, such as a higher surface area, faster kinetics due to short percolation pathways and large free volume, while this is not the case with crystalline host structures, whose performance depends on various factors, such as structural stability, crystal orientation, phase transition, and limited sites for the incoming guest ions.^[^ ^37^, ^38^ ^]^ The FT‐IR spectrum (Figure 1b) and Raman spectrum (Figure 1c) show the bands for the as‐synthesized PVMPO powder. Figure 1d, e shows the morphology of as‐synthesized PVMPO at lower and higher magnifications. PVMPO shows non‐uniform morphology and non‐uniform particle size. The pores can be seen on the polymer, which helps in better electrolyte wettability.

### 4.2 Rheology, Adhesion Strength, and TPC Measurements

**Figure 2:**
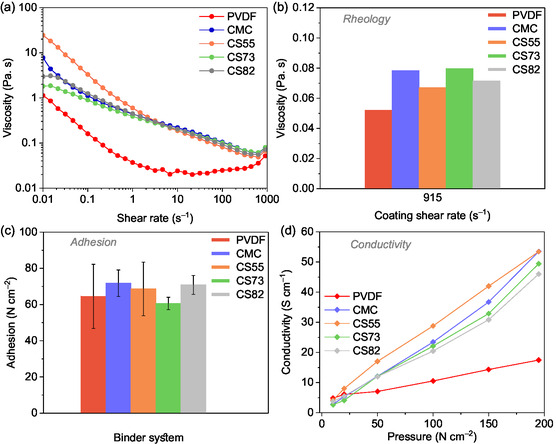
Rheological properties of the electrode pastes, showing ideal battery electrode paste behavior (average of three different measurements for each electrode paste), are presented. a) Viscosity is shown as a function of shear rate, b) a bar graph showing the viscosity of the respective electrode pastes at the coating shear rate of 915 s^−1^, c) adhesion strength measurements of the not‐pressed electrodes processed with different binders (error bars correspond to the standard deviation of three different measurements), and d) TPC measurements of the same are included.

The rheology of the prepared electrode pastes greatly influences the coating process as well as the electrode microstructure.^[^ ^39^ ^]^ The viscosity of the electrode pastes was investigated to determine the flow behavior of the electrode paste. Here, the viscosity of primary (PVdF, CMC) and binary binder systems (CMC + SBR) was studied. **Figure** **2**a shows the averaged plot of the three measurements for each binder system. An ideal battery electrode paste should show strong non‐Newtonian fluid behavior, i.e., shear thinning with an increase in the shear rate.^[^ ^24^, ^25^, ^40^ ^]^ A higher viscosity of the electrode paste at the lower shear rate would stabilize the electrode paste, while a lower viscosity at higher shear rate enables homogeneous mixing and deagglomeration during casting.^[^ ^41^ ^]^ All investigated binder systems show the non‐Newtonian fluid behavior as the electrode paste viscosity decreases with increased shear rate (Figure 2a). Thus, the electrode pastes prepared with different aqueous binders at different compositions show similar behavior to that of the reference PVdF‐based electrode paste.

All electrode pastes were cast on the current collector at a coating speed of 50 mm s^−1^, and the gap width of 50 μm. Thus, at this coating speed and the gap width maintained, the shear rate was found to be 1000 s^−1^. However, this value was not measured by the instrument. Instead, the value closest to 1000 s^−1^, which is 915 s^−1^, was considered. The bar graph (Figure 2b) shows the viscosity of the electrode pastes at the coating shear rate of 915 s^−1^ (close to 1000 s^−1^). All aqueous systems show higher viscosity at the coating shear rate compared to the PVdF system (Figure 2b). The higher viscosity of the water‐based electrode pastes was mainly attributed to the thickening properties of CMC at the corresponding weight ratios. CMC is the thickening agent, and the addition of the elastomer (SBR) did not have a notable influence on the flow behavior, as well as the electrode paste viscosity at the coating shear rate. The variation in viscosity did not affect the performance of the aqueous‐processed electrodes.

The adhesion strength is defined by the maximum force needed to separate the coating from the current collector, and it strongly depends on the binder used. It is an important physical electrode property, as the coating must maintain mechanical integrity during cycling as well as during the cell processing steps. The adhesion of the electrodes is determined by pull‐off strength measurements as proposed by Haselrieder et al.^[^ ^42^ ^]^

The adhesion strength of the dried electrode sheets (not‐pressed) prepared using five different binder systems was determined and is shown in Figure 2c. CMC‐based electrode exhibits higher adhesion strength compared to other binder systems. Beyond that, the water‐processed electrodes demonstrate a higher or comparable adhesion to the PVdF‐based electrode. The addition of higher amounts of SBR reduces the adhesion strength (Figure 2c). This well aligns with the fact that CMC provides the necessary adhesion to the current collector. The lower adhesion of PVdF‐based electrode might stem from the indirect binding nature, unlike water‐processable binders. Additionally, PVdF dissolves during processing, leading to different structural and mechanical characteristics. However, in other cell chemistries, PVdF consistently exhibits superior mechanical properties compared to CMC‐based binder systems.

The TPC was determined for dried electrode sheets (not‐pressed) prepared using five different binder systems. Figure 2d shows the TPC of the electrodes at different applied forces. The force applied varies from 10 N to 50 N, 100 N, 150 N, and 195 N, and returns to 100 N with holding time of 20 s for each step. The highest pressure of 195 N corresponds to the internal pressure of a CR2032 coin cell.^[^ ^43^ ^]^ However, the internal pressure of the Swagelok cell setup remains unknown. So, the linear plot for all applied pressures is presented. A linear increase in the conductivity is noticed with increasing applied pressure (Figure 2d). The aqueous‐processed electrodes show higher electronic conductivity of 53 S cm^−1^ (CMC and CS55 electrodes), 49 S cm^−1^ (CS73), and 46 S cm^−1^ (CS82) than PVdF with a lower conductivity (17 S cm^−1^) at 195 N. The added conductive additive wraps the polymer properly, forming a porous conductive network, resulting in improved conductivity. Aqueous‐processed electrodes exhibit enhanced conductivity despite the presence of inhomogeneities. The lower conductivity value of the PVdF electrode can be associated with the insulating nature of PVdF.^[^ ^21^ ^]^ As previously mentioned, it could be attributed to variations in the electrode microstructure caused by polymer dissolution during processing with PVdF/NMP.

**Figure 3:**
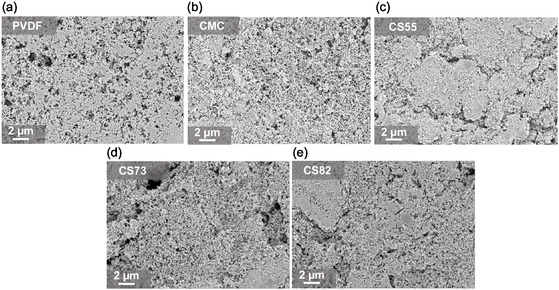
Surface morphology images from SEM analysis of pristine electrodes with different binder systems at 5kx magnification: a) PVdF, b) CMC, c) CS55, d) CS73, and e) CS82 electrodes.

The surface morphology of the pristine electrodes for the all five‐binder system is shown in **Figure** **3**a–e. Figure 3a shows the morphology of the electrode fabricated with PVdF binder. The PVdF‐based electrode shows a homogeneous surface morphology without indications of agglomerates or delamination effects. The reason for the absence of PVMPO particles in the PVdF‐based electrode is because of the solubility of PVMPO in NMP, thereby providing a more homogeneous electrode with a porous, electronically conductive network. In contrast, with aqueously processed electrodes, the polymer is not soluble in water, and carbon black covers most of the polymer along with the binder.

CMC‐based electrode has shown a more uniform homogeneous morphology similar to that of the PVdF‐based electrode (Figure 3b). However, more pronounced and larger agglomerations in the other three systems, CS55, CS73, and CS82, were observed (Figure 3c–e). It is observed that the addition of SBR leads to agglomerations. The polymeric binders are not visible on the SEM images, which are likely to coat the active material and the conductive carbon. The backbone of all solid components in the electrode is made of carbon and is quite challenging to differentiate in the SEM image. However, elemental mapping would greatly help to identify the distribution. EDS mapping on the pristine electrodes was performed and the results are shown in Figure S1, Supporting Information. The distribution of PVMPO (active material) can be identified by the nitrogen heteroatom as it is present in the phenoxazine redox moiety (Figure 1a, inset). Elemental mapping images of all electrodes show the uniform distribution of the polymer, conductive additive, and binder.

To sum up, water‐based binder systems provide better adhesion and electronic conductivity compared to PVdF‐based systems, making them a potential alternative for sustainable processing. However, agglomerations remain a challenge that can be addressed using high‐energy mixers that do not damage the active material. The reason behind the lower adhesion and conductivity of PVdF‐based electrodes might be due to different electrode microstructures, but the exact reason remains unclear.

### 4.3 Electrochemical Performance

**Figure 4:**
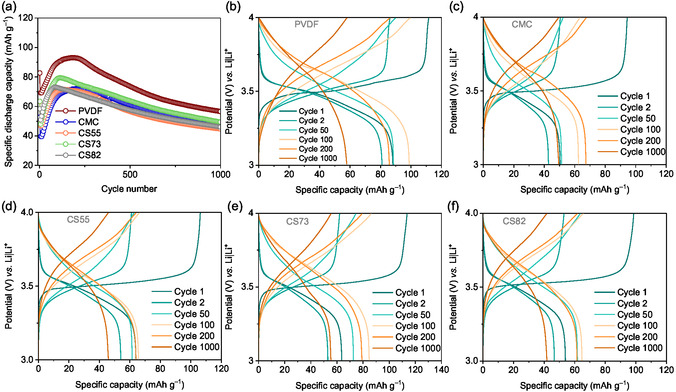
Electrochemical performance of PVMPO‐electrode using different binders: a) Comparison of the cyclic stability for 1,000 cycles at 1C rate (the graph shown is the average of three measurements for each system, and the corresponding standard deviation is shown in Figure S2, Supporting Information). b) Potential *vs.* capacity profiles of all five‐binder systems for selected cycles corresponding to a single cell: (b) PVdF, c) CMC, d) CS55, e) CS73, and f) CS82.

In this study, electrodes processed with PVdF binder are used as a reference to compare the performance with aqueous‐processed electrodes. The electrochemical investigation was done with constant current cycling (CCC) at a 1C rate. **Figure** **4**a shows the specific discharge capacity (mean of the long‐term cycling stability of three cells) for each binder system. From the cycling stability plot, almost all binder systems exhibit a similar performance. PVdF‐based electrode shows a slightly higher capacity at the end of 1,000 cycles (56 mAh g^−1^) compared to other aqueous‐processed electrodes. However, considering capacity retention, the CMC‐based electrode shows 92% capacity retention at the end of 1,000 cycles while compared to other investigated binder systems. The potential *vs.* capacity profile for electrodes based on all binder systems at the selected cycles are shown in Figure 4b–f. From the potential *vs.* capacity profile for the electrodes made using PVdF (Figure 4b), it can be clearly seen that the initial discharge capacity is found to be 88 mAh g^−1^ followed by a gradual decrease in capacity. From the 50^th^ cycle, the capacity gradually increases and reaches a maximum value around 200 cycles (93 mAh g^−1^) followed by a steady decrease in capacity, resulting in 68% of capacity retention at the end of 1,000 cycles. A similar phenomenon is observed for all aqueous‐processed electrodes (Figure 4c–f) with 50% capacity accessible in the first cycle followed by an increase in capacity till 200 cycles, and at the end of 1,000 cycles, the resulting capacity is found to be 50% of the theoretical specific capacity of PVMPO (120 mAh g^−1^) which well supports the phenomena of dissolution and deposition occurring in all binder systems.^[^ ^17^ ^]^ It is also clear that the potential *vs.* capacity profile is more sloped from 100 cycles with an increase in the nominal electrode potential (3.62 V *vs.* Li|Li^+^), while in the initial cycle it is 3.49 V *vs.* Li|Li^+^. The slopy profile hints at a less defined redox process occurring in the electrode after a few hundred cycles.^[^ ^17^ ^]^ From these results, it is evident that deposition and dissolution phenomena occur during the cycling of PVMPO. The initial decrease in capacity could be explained by the dissolution of the oxidized form of PVMPO in the electrolyte and an incomplete reduction back to neutral PVMPO. The maximum capacity is achieved at around 200 cycles, followed by a capacity fade. However, Otteny et al. have achieved the maximum capacity after ≈700 cycles.^[^ ^17^ ^]^

Figure S2, Supporting Information, presents the standard deviation plot of all five investigated binder systems. The CMC and CS55 systems show a lower standard deviation, while CS73 and CS82 show higher deviation values. The higher deviation is caused by the non‐homogeneity in the electrodes upon the addition of SBR. Figure S3, Supporting Information, shows the photograph of a CMC electrode cycled at 1C rate after 10,000 cycles. The intact electrode indicates strong adhesion of the solid components of the composite electrode to the current collector. The capacity retention of all investigated binder systems at the end of 1,000th cycle is shown in Table S1, Supporting Information.

In summary, the performance of the aqueous binder systems is quite similar to that of the PVdF‐based system, with a difference in electrode capacity of 6 to 10 mAh g^−1^ at the end of 1,000 cycles. However, 92% capacity retention at the end of 1,000 cycles is observed for the CMC‐based composite electrodes, while the PVdF systems delivered 68% capacity retention. Integrating water‐soluble binders strongly complements the sustainability profile of battery systems. Thus, it can be concluded that the CMC‐based binder system is a possible binder to be used in the electrode processing of PVMPO. In the following, CMC‐based electrode (without SBR addition) are used for further electrochemical investigation.

### 4.4 Optimizing Electrolyte Composition to Access the Maximum Electrode Capacity

Due to the dissolution of the oxidized state of PVMPO in the electrolyte, its accessible specific capacity was reduced. Oxidized PVMPO is soluble in 1.0 m LiPF_6_ in a 1:1 EC:DMC mixture (referred to as DMC‐based). Therefore, 1.0 m LiPF_6_ in a 3:7 EC:EMC mixture (referred to as EMC‐based), which inhibited dissolution in PVMPT,^[^ ^27^ ^]^ is herein investigated for PVMPO.

**Figure 5:**
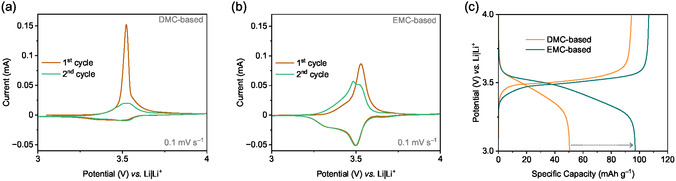
Cyclic voltammograms at 0.1 mV s^−1^: a) DMC‐based and b) EMC‐based. c) Potential *vs.* capacity profiles showing 1st (dis)charge using DMC‐ and EMC‐based electrolytes at a 1C rate.

CV enables the identification of the polymer's solubility into the electrolyte by comparing the current values in the initial cycles. **Figure** **5**a, b displays the CVs of the first two cycles performed at a scan rate of 0.1 mV s^−1^ with different electrolytes. The CVs of the PVMPO composite electrode with two different electrolytes (DMC‐based and EMC‐based) has shown reversible redox reactions. The first cycle oxidation of PVMPO occurs at 3.52 V *vs.* Li|Li^+^, and the corresponding reduction occurs around 3.34–3.53 V *vs.* Li|Li^+^ and for the second cycle, the oxidation occurred at 3.5 V *vs.* Li|Li^+^ when a DMC‐based electrolyte is used. In case of EMC‐based electrolyte, the first cycle oxidation occurs at 3.53 V *vs.* Li|Li^+^, and the corresponding reduction occurs around 3.3–3.5 V *vs.* Li|Li^+^, and the second cycle oxidation occurred at around 3.48 V to 3.5 V *vs.* Li|Li^+^. The first oxidation (1st charge) occurred at a higher current, indicating an increase in ionic/electronic conductivity upon oxidation and structural rearrangement of the polymer due to the insertion of anions from the electrolyte for charge compensation in both DMC‐based and EMC‐based electrolytes.^[^ ^17^ ^]^ However, in the corresponding reduction process, the oxidized polymer is soluble in the DMC‐based electrolyte, resulting in poor reversibility and lower current values, which hints at the dissolution of the oxidized state in the electrolyte and an incomplete reduction back to the neutral state. On the contrary, the EMC‐based electrolyte exhibits better reversibility. Considering the current values obtained in the second cycle for both electrolytes, it is evident that the current value for the EMC‐based electrolyte during oxidation (charge) and reduction (discharge) is 0.056 mA and 0.05 mA, respectively. In contrast, for the DMC‐based electrolyte, the discharge current (0.001 mA) is lower than the charge current (0.02 mA). The current values obtained are considered to correlate with the dissolution behavior of the active material.^[^ ^27^ ^]^ The lower discharge current value for the DMC‐based electrolyte suggests the solubility of the oxidized polymer and an incomplete reduction of the oxidized polymer to the neutral state upon discharge. However, in the EMC‐based electrolyte system, the current values remain higher than those in the DMC‐based one, and the discharge current values remain similar, implying inhibition of polymer dissolution. The peak splitting observed for the EMC‐based electrolyte in the second cycle likely results from the insertion of anions into the polymer structure.

Figure 5c shows that with the DMC‐based electrolyte, upon charging to 4 V, the first specific charge capacity achieved is around 95 mAh g^−1^, while the specific discharge capacity achieved is around 51 mAh g^−1^. This corresponds to only 54% of the Coulombic efficiency, which again supports the assumption of dissolution of oxidized PVMPO in the charged state. When the EMC‐based electrolyte is used, the first specific charge capacity achieved is around 105 mAh g^−1^ and the specific discharge capacity achieved is around 97 mAh g^−1^, corresponding to a Coulombic efficiency of 92%. As evident from the CVs, this confirms that the EMC‐based electrolyte effectively suppresses the dissolution of oxidized PVMPO.

In the DMC‐based electrolyte (1:1 EC:DMC), the higher proportion of EC in the solvent mixture enhances solubility and facilitates the dissolution of conductive salts, indicating good polymer solubility in the electrolyte. Consequently, introducing a methyl group to DMC (forming EMC) and reducing the EC concentration leads to lower viscosity, and the incorporation of a bulkier group influences the solvent‐polymer interactions. The relative permittivity of the solvent mixture collectively reduces solubility while improving reversibility.^[^ ^27^ ^]^

**Figure 6:**
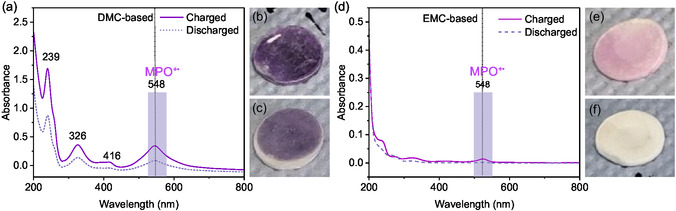
UV/Vis‐spectroscopic measurements of electrolyte extracted from separators of (dis)charged cells of PVMPO composite electrodes: a) DMC‐based electrolyte; b,c) Photograph of the charged and discharged separator (DMC‐based); d) EMC‐based electrolyte; e,f) Photograph of the charged and discharged separator (EMC‐based), respectively.

To confirm the dissolution of oxidized PVMPO as well as its inhibition, UV/Vis‐spectroscopic analysis was performed by extracting the electrolyte from the separators after initial cycles. **Figure** **6** shows the corresponding spectra for both electrolyte systems, as well as photographs of the separators in the charged and discharged states. The charged cells of the DMC‐based electrolyte have shown the presence of oxidized phenoxazine units (548 nm) in the electrolyte (Figure 6a). Upon discharge, the intensity decreases, but the peak remains visible, which implies that not all dissolved oxidized polymer is deposited back on the electrode surface upon discharge. The dark purple‐colored separator of the charged cell (Figure 6b) further indicates the dissolution of oxidized PVMPO, while in the discharged separator, only a mild purple color is observed (Figure 6c). This implies the incomplete reduction of charged PVMPO back to the neutral polymer. In contrast, when using the EMC‐based electrolyte, the intensities of the peaks of oxidized PVMPO are much lower in the charged states, indicating negligible dissolution. Upon discharge, no bands are observed in the UV/Vis spectra (Figure 6d). Figure 6e, f shows photographs of the separator in the charged and discharged state from the EMC‐based electrolyte. The pale purple color indicates a minor amount of dissolved oxidized polymer, and the colorless separator upon discharge shows that the dissolved polymer was completely reduced back to neutral PVMPO during discharge.

**Figure 7:**
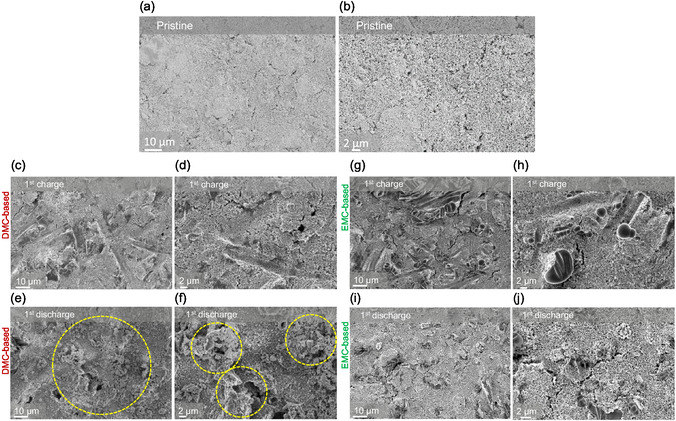
SEM images of the a,b) Pristine electrode; (dis)charged electrodes with the c–f) DMC‐based and g–j) EMC‐based electrolyte.

The dissolution and deposition mechanism in the initial cycle was further investigated by SEM to understand the (dis)charge mechanism. **Figure** **7**a, b shows the morphology of a pristine PVMPO composite electrode. Figure 7c–f shows the morphology of the electrode surfaces after 1st charge and 1st discharge while using the DMC‐based electrolyte. During the first charge, PVMPO is oxidized and dissolves in the electrolyte, and upon discharge, the dissolved oxidized states re‐deposit on the surface of the electrode. The deposited PVMPO on the discharged electrode in the first cycle can be seen in the SEM image (dotted yellow circles). While using the EMC‐based electrolyte, no deposition is observed upon discharge, (Figure 7g–j) as no dissolution takes place during charge. The elemental mapping of these charged and discharged electrodes while using DMC‐and EMC‐based electrolyte is shown in Figure S4, Supporting Information.

**Figure 8:**
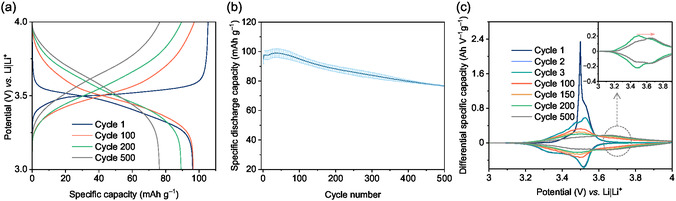
Electrochemical performance when cycled at 1C rate with EMC‐based electrolyte a) Potential *vs.* capacity profile, b) Cycling stability of three cells cycled for 500 cycles with error bars, c) Differential capacity *vs.* potential plot of selected cycles for the cell operated at 1C rate with inset showing the enlarged view showing the peak shift over the cycles.

CCC investigations were performed to assess the long‐term stability of the PVMPO composite electrode when cycled at a 1C rate with EMC‐based electrolyte. The potential *vs.* capacity with selected cycles cycled at 1C rate is shown in **Figure** **8**a. Figure 8b shows the long‐term cycling stability with a standard deviation of three measurements. The 1st specific discharge capacity achieved is 97 mAh g^−1^ followed by a slight increase to 100 mAh g^−1^, and is almost stable during 100 cycles (96 mAh g^−1^), which corresponds to ≈80% of the theoretical specific capacity (120 mAh g^−1^). However, there is a steady capacity fading after 100 cycles to 500 cycles to 77 mAh g^−1^ which corresponds to 64% of the theoretical specific capacity. However, the capacity retention at the end of 500 cycles, taking the 1st cycle discharge capacity is found to be 79%. After 100 cycles, the potential profile becomes slopy, and the discharge potential increases to 3.61 V *vs.* Li|Li^+^ (at the end of 500 cycles). This increased potential and slopy potential *vs.* capacity profiles hint at low redox activity in the polymer^[^ ^17^ ^]^ and, thus, a decrease in the capacity (Figure 8a). Table S2, Supporting Information, shows the percentage of capacity retention at different cycles.

**Figure 9:**
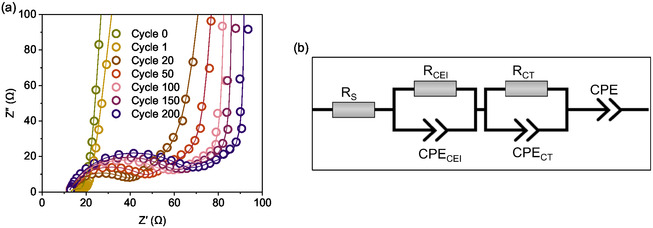
a) PEIS spectra of the cell operated at a 1C rate after selected cycles. Experimental data points are represented by legends, while the fitted data is shown as lines passing through these symbols. b) Represents the equivalent circuit model used for the fitting.

The differential capacity *vs.* potential plot provides insights into cell behavior and performance. Figure 8c presents the differential specific capacity *vs.* potential plot derived from the long‐term cycling stability data. The first high intensity peak at 3.5 V can be attributed to electrolyte oxidation and the insertion of anions into the polymer structure. A slight shift in oxidation potential is observed, increasing from 3.5 V *vs.* Li|Li^+^ in the first cycle to 3.52 V *vs.* Li|Li^+^ in the second and third cycles. As cycling progresses, the peak intensity gradually decreases, accompanied by changes in peak shape. This reduction in intensity corresponds to the observed capacity loss. Additionally, peak broadening is evident, indicating decreased ion kinetics. A noticeable peak shift toward higher potential occurs after 200 cycles, increasing from 3.49 V to 3.65 V in the subsequent cycles (Figure 8c, inset). This redox shift to higher potential suggests an increase in cell resistance over cycling, which aligns with impedance measurements (**Figure** **9**). These findings indicate that, over repeated cycles, the internal resistance of the cell increases, leading to reduced kinetics and fewer redox reactions.

Figure 9 presents the Nyquist plot obtained after (OCV) open circuit voltage and subsequent cycles. The plot comprises several components: R_S_ solution resistance (high frequency region), a semicircle in the mid frequency region, which shall be attributed to R_CEI_ – interfacial/interphasial resistance and R_CT_ – charge transfer resistance, and a slope in the low frequency region, which is modeled as a constant phase element.^[^ ^44^, ^45^ ^]^ R_S_ remains almost identical upon cycling. However, the size of the semicircle noticeably increased (Figure 9a). The resistance values for these components are presented in **Table** **2**, while Figure 9b shows the equivalent circuit used for fitting.

**Table 2:** Resistance values of each component, as obtained from the EIS spectra fitting parameters for the data presented in Figure 9. *(R*_*S*_ – *solution resistance; R*_*CEI*_ *– interfacial/interphasial resistance; R*_*CT*_ *– charge transfer resistance)*.

Cycle/Condition	R_S_ (Ω)	R_CEI_ (Ω)	R_CT_ (Ω)
Cycle 0	13	5.34	3.84
Cycle 1	12.6	8.01	2.03
Cycle 20	12.1	27.5	26.9
Cycle 50	12.2	35.7	25.6
Cycle 100	12.3	45.5	24
Cycle 150	12.3	49.1	25.4
Cycle 200	12.4	54.6	25.8

After OCV, a depressed semicircle appears. Shortly after the first cycle, the R_CT_ drops to 2.03 Ω, showing improved ionic conductivity. However, the R_CEI_ resistance continues to rise with subsequent cycling, suggesting that the CEI layer becomes thicker. The overall resistance of the cell (R_S_ + R_CEI_ + R_CT_) increases upon cycling, contributing to a lowered ionic conductivity. Thus, the increased cell resistance results in a higher overpotential, leading to capacity decay.

**Figure 10:**
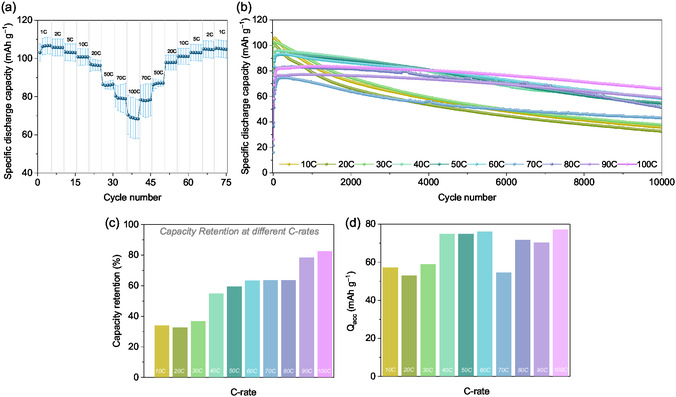
High‐rate performance of PVMPO composite electrode in the EMC‐based electrolyte a) Rate capability b) Comparison of the cycling stability at different current rates (0.26–0.29 mg cm^−2^) c) Capacity retention at various C‐rates, calculated by considering the specific discharge capacities at the 65^th^ cycle and at the end of 10,000 cycles and d) bar graph showing the accumulated discharge capacity at different C‐rates.

Further, the rate performance of the PVMPO composite electrodes at elevated C‐rates was investigated. Here, the cells were cycled at different current rates with a 12 h rest time after the cell assembly. **Figure** **10**a shows the rate capability of the PVMPO composite electrode from 1C to 100C for 75 cycles (standard deviation with two different measurements). It demonstrates excellent rate capability comparable to that of PVMPT.^[^ ^46^ ^]^ The capacity achieved at 1C rate for the first cycle was 103 mAh g^−1^, but while cycling at 100C, the capacity achieved was 70 mAh g^−1^. However, upon decreasing the current rate sequentially to 1C, the capacity achieved after cycling at different C‐rates for 70 cycles was 105 mAh g^−1^. This is akin to all other C‐rates. It can be concluded that the polymer remains quite stable even after cycling at elevated current rates.

Further, the long‐term cycling performance at higher current rates (from 1C to 100C) for 10,000 charge/discharge cycles has been investigated (Figure 10b). The corresponding standard deviation plot is shown in Figure S5, Supporting Information. For rates of 10C, 20C, and 30C, the first cycle capacities achieved were 82 mAh g^−1^, 77 mAh g^−1^, and 67 mAh g^−1^, respectively. This was followed by an increase in capacity over the next hundred cycles, after which the capacity slowly and steadily decreased, resulting in 36 mAh g^−1^, 33 mAh g^−1^, and 38 mAh g^−1^ corresponding to 44%, 42% and 57% capacity retention after 10,000 charge/discharge cycles, respectively. However, the cells cycled at 40C to 100C showed lower initial capacities (between 25 and 36 mAh g^−1^) followed by a steady increase in the capacity over a few hundred cycles. Notably, cycling at 100 °C demonstrated a better cycling stability with 82% capacity retention after 10,000 cycles (66 mAh g^−1^).

Figure 10c presents the capacity retention at the end of 10,000 cycles. The capacity retention at the end of 10,000 cycles was determined by taking into account the specific discharge capacity of the 65th cycle, as the capacity for all C‐rates stabilizes at this point, and the specific discharge capacity at the end of 10,000 cycles. The accumulated specific discharge capacities (Q_acc_) were calculated for each C‐rate to quantify the shuttled specific discharge capacity. Figure 10d shows the plot of Q_acc_ for all C‐rates for 10,000 cycles. The higher capacity shuttle is observed when cycled at 100C, which again supports the high‐rate capability of PVMPO.

The faster kinetics of PVMPO can be attributed to its planar geometry. According to semiclassical electron‐transfer theory and its extensions, electronic coupling and reorganization energy are the two major parameters that govern self‐exchange electron and charge transfer in π–conjugated systems. The reorganization energy should be as low as possible for efficient charge transport. This reorganization energy is determined by the molecular geometry of the redox‐active unit in both its neutral and charged states.^[^ ^47^ ^]^ In the case of phenoxazine, the planarization angle is larger (155°) in the neutral state and changes to 174° in the radical cationic state (charged state), which is a smaller change compared to phenothiazine (138° in the neutral state and 164° in the radical cationic state).^[^ ^17^ ^]^ Furthermore, the influence of π–π stacking in phenoxazine is lower than in phenothiazine, making the phenoxazine units more accessible to charge‐balancing counter anions. Therefore, the lower reorganization energy and reduced influence of π–π stacking interactions between the phenoxazine moieties result in an excellent intrinsic charge mobility. In short, the planar conformation of the phenoxazine redox unit in both neutral and cationic states contributes to faster charge‐transfer kinetics, enabling higher C‐rates of up to 100C with excellent capacity retention.

### 4.5 Investigation of Possible Degradation Mechanism

Despite the good cycling stability, a decline in capacity over multiple cycles is observed. Therefore, the underlying reasons or possible mechanisms behind capacity fading are investigated. First, the Li metal was replaced after 200 cycles, assuming dendrite formation as a possible reason for capacity fading as a black layer was observed on the lithium surface (Figure S7b–d–inset, Supporting Information). Figure S6a, Supporting Information, shows the cyclic stability plot with an indication for the lithium replacement after 200 cycles. The cycled electrode was not washed after disassembly. The assembled cell was given a rest time of 6 h and cycled again, where no change in the cycling behavior was observed, implying no dendrite formation on the lithium side. Figure S6b, Supporting Information, shows the potential *vs.* capacity profile for the selected cycles before lithium replacement, and Figure S6c, Supporting Information shows the data for 201st, 250th, 300th, and 400th cycles. It is worth noticing that no changes were observed in the potential versus capacity profiles with the Li replacement process showing high similarity to Figure 8a. Thus, no lithium dendrite formation occurs, which was also confirmed by *ex situ* SEM measurements (Figure S7, Supporting Information) of cycled lithium foil, which hints at the possibility of a different mechanism for capacity fading.

**Figure 11:**
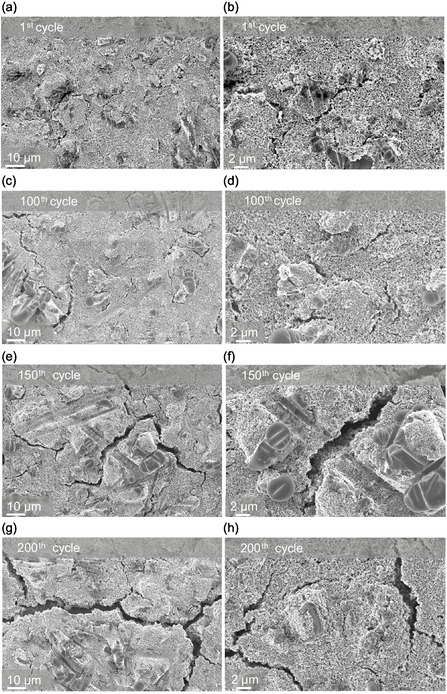
*Ex situ* SEM images of cycled electrodes after specific cycles with EMC‐based electrolyte at lower and higher magnifications, respectively, a,b) 1st cycle, c,d) 100th cycle e,f) 150th cycle and g,h) 200th cycle, showing the formation of cracks on the electrode surface.

**Figure** **11** shows the *ex situ* SEM images of the cycled electrodes after 1st, 100th, 150th, and 200th cycles. As seen in the SEM image, cracks develop in the electrode over the cycles. The electrode components remain intact up to 100 cycles, while large deep canals and cracks are observed upon further cycling. Thus, crack formation leads to a contact loss of active material, and hence, the capacity fades after 100 cycles. The corresponding EDS image is shown in Figure S8, Supporting Information.

**Figure 12:**
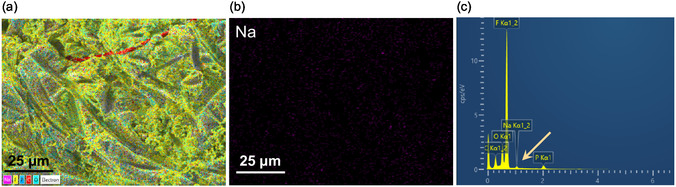
a) EDS mapping of the lithium metal surface after 500 cycles, b) corresponding EDS signal highlighting sodium (magenta), and c) Spectrum of the mapped area. Other detected elements are represented as follows: fluorine (F) in yellow, phosphorus (P) in blue, carbon (C) in red, and oxygen (O) in cyan.

**Figure** **12** displays EDS mapping images of elements detected on the lithium surface after 500 cycles, along with the corresponding sodium distribution and spectrum. The presence of fluorine, phosphorus, and carbon is likely attributed to the decomposition of the electrolyte. In contrast, the detection of sodium (Na) suggests degradation of the PVMPO composite electrode, specifically the Na‐CMC binder. Oxygen may originate from the PVMPO, the electrolyte, or the binder. The EDS images also indicate the presence of carbonates and other decomposition products. While polymer residues are not visibly detected on the lithium counter electrode, the presence of sodium further supports the breakdown of the PVMPO composite electrode. It is also possible that nitrogen may be present in the separator.

Figure S7, Supporting Information, shows the SEM images of pristine lithium and cycled lithium at 1C rate after 100, 150, and 200 cycles, and the inset shows the image of the cycled lithium counter electrode where a black layer was noticed on the surface of the lithium. SEM images with indications reveal the deposits of the electrode and carbonates (from the electrolyte). To further verify the presence of PVMPO on the cycled lithium surface, *ex situ* FT‐IR measurements were conducted on the lithium metal after 200 cycles. While bands corresponding to the electrolyte are visible, identifying bands related to the decomposed polymer is challenging due to the absence of distinct functional groups in the polymer. (Figure S9, Supporting Information).

In summary, the EMC‐based electrolyte effectively suppresses the dissolution of the oxidized PVMPO. However, as cycling progresses, the capacity gradually declines, likely due to the degradation of the polymer electrode. This degradation may be linked to the weaker π–π interactions between the redox‐active moieties in PVMPO as reported by Otteny *et al*.^[^ ^17^ ^]^

## 5 Charge Storage Kinetics of the PVMPO Electrodes

Furthermore, electrochemical kinetic studies were carried out to determine the charge storage mechanism in the PVMPO electrode. CV is a powerful tool to quantify the nature of the reaction, such as surface‐controlled reactions and diffusion‐controlled reactions.^[^ ^48^ ^]^ Dunn and co‐workers proposed the quantitative deconvolution of pseudocapacitance and diffusion contribution of the redox reaction from CV.^[^ ^49^, ^50^ ^]^

**Figure 13:**
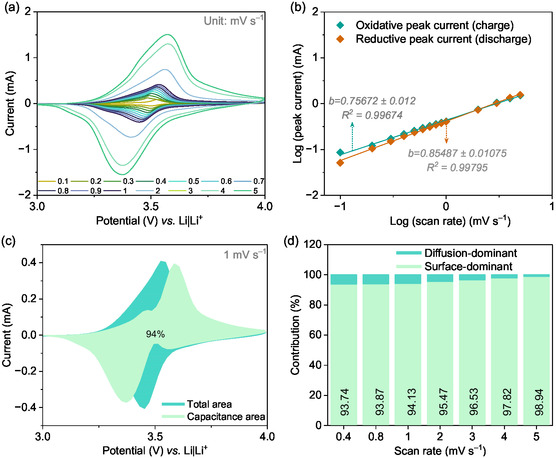
Electrochemical kinetic study: a) CV curves of PVMPO composite electrodes at different scan rates. b) Determination of b value from the peak currents c) Capacitive contribution of the redox reaction in the CV at 1 mV s^−1^ d) Capacitance and diffusion contributions of the redox reaction at different scan rates.

**Figure** **13**a shows the typical CVs for the PVMPO composite electrode at various scan rates. The area under the curve provides the total charge stored as a result of Faradaic and non‐Faradaic processes. The oxidative and reductive peak represents the anion (PF_6_^−^) insertion (charge) and de‐insertion (discharge). The total charge stored depends on the scan rate in the range between 0.1 mV s^−1^ and 5 mV s^−1^. The total charge stored is the combination of two different processes: the Faradaic contribution from ion insertion and charge transfer with surface atoms (pseudocapacitance) and the non‐Faradaic contribution from the double layer effect. It can be clearly seen from the scan‐rate dependent CVs that the peak currents (*i*) increase with scan rate (*v*). Herein, the measured current obeys the power law relationship with the scan rate (*v*), which leads to

$$i=av^{b}$$

Here, *a* and *b* are adjustable parameters where the b value can be obtained by the slope of the plot of log *i* *vs.* log *v*. Here, the *b* value of 0.5 indicates the diffusion‐dominant redox processes while the *b* value of 1 indicates surface‐dominant redox processes. From the linear fit plot of log (peak current) *vs.* log (scan rate), the *b* value obtained is 0.757±0.012 for anodic sweep and 0.855±0.011 for cathodic sweep (Figure 13b). This suggests that the reaction at the PVMPO electrodes is primarily governed by surface‐dominated phenomena, with a minimal contribution from diffusion processes.

The closer examination of the CV helps to quantitatively distinguish the surface dominant and diffusion dominant contributions to the overall current response. In this study, Dunn's method is to deconvolute the surface and bulk phenomena from the equation^[^ ^49^ ^]^

$$i\left(V\right)=k_{1}v+k_{2}v^{1/2}$$

Here $k_{1}v$ and $k_{2}v^{1/2}$ corresponds to the current obtained from surface dominant and diffusion dominant processes, respectively. When rearranged, this equation can be written as

$$\frac{i\left(V\right)}{v^{1/2}}=k_{1}v^{1/2}+k_{2}$$

The plot of $\frac{i\left(V\right)}{v^{1/2}}$ can be plotted as a function of $v^{1/2}$. The linear fit of this provides the slope and intercept, which corresponds to $k_{1}$ and $k_{2,}$ respectively.

In this study, 10 data points were collected at each scan rate and used for the calculations. Figure 13c compares the measured capacitance current with the total current, with the shaded area highlighting the capacitance current at a scan rate of 1 mV s^−1^. From Figure 13d, it is clear that the redox processes in the PVMPO electrodes are predominantly surface‐controlled, accounting for 94% to 99% of the total activity at scan rates between 0.1 and 5 mV s^−1^. This behavior is likely due to the planar structure and amorphous nature of the PVMPO (as shown in Figure 1a), which provides shorter percolation paths for incoming anions, leading to faster reaction kinetics. Additionally, the porous structure of the conductive additive in the electrode shortens the diffusion distance between the electrolyte and the redox sites, further enhancing the speed of the redox processes. This combination of factors also explains the polymer's high‐rate performance, as the minimized diffusion path lengths allow for rapid ion movement.

## 6 Conclusion

In summary, the aqueous processing of a *p*‐type PVMPO polymer active material for positive electrodes in lithium‐organic batteries with a discharge potential of 3.52 V *vs.* Li|Li^+^ was investigated. According to our knowledge, this is the first report with special emphasis on the aqueous processing of an organic redox polymer. Among the electrodes using aqueous binders, the PVMPO‐CMC composite exhibited the best performance, retaining 92% of its capacity after 1,000 cycles. A challenge with PVMPO is the dissolution of the oxidized state into the electrolyte, resulting in capacity decay. This dissolution could be effectively inhibited by using 1.0 m LiPF_6_ in 3:7 EC:EMC electrolyte, resulting in 79% capacity retention at the end of 500 cycles. UV/Vis‐ spectroscopic and *post* cycling SEM measurements confirmed the inhibition of dissolution. The rate performance of the PVMPO composite electrodes was investigated at elevated C‐rates, showcasing 82% capacity retention at the end of 10,000 cycles at 100 C. This rapid kinetics can be attributed to the planar structure of phenoxazine as well as the amorphous nature of the phenoxazine polymer, providing shorter percolation paths and enabling faster kinetics, which is also deconvoluted by scan‐rate dependent cyclic voltammetry. The *post *cycling characterization of the electrodes cycled at 1C using SEM revealed that PVMPO electrodes developed cracks after 100 cycles, resulting in loss of active material and thereby decreased capacity. Additionally, a black layer was formed on the lithium counter electrode, which was further confirmed as deposition of electrolyte as well as electrode decomposition products by EDS and FT‐IR measurements, providing insights into the capacity fading mechanism of the phenoxazine polymer. This study underscores the aqueous processing of PVMPO and dissolution inhibition, which not only enhanced overall electrochemical performance but also reduced the environmental impact by transitioning to water‐based processing.

## Conflict of Interest

The authors declare no conflict of interest.

## Supporting information

Supplementary Material

## Data Availability

The data that support the findings of this study are available from the corresponding author upon reasonable request.
