# Supplementary material for: From Fabrication to Failure—Aqueous Processing, Electrolyte Tuning, and Degradation Mechanism Elucidation in Poly(3‐Vinyl‐N‐Methylphenoxazine) Positive Electrodes
Source: ChemSusChem. 2025 Aug 10;18(19):e202500753. doi: 10.1002/cssc.202500753 (PMC12487756; doi:10.1002/cssc.202500753)
Supplement: Supplementary file 1 — Supplementary Material [file CSSC-18-e202500753-s001.pdf]

Supporting information

From Fabrication to Failure—Aqueous Processing, Electrolyte Tuning and Degradation Mechanism Elucidation in Poly(3-vinyl-*N*-methylphenoxazine) positive electrodes

Sathiya Priya Panjalingam, Somayeh Ahadi, Markus Börner, Birgit Esser, Martin Winter and Peter Bieker*

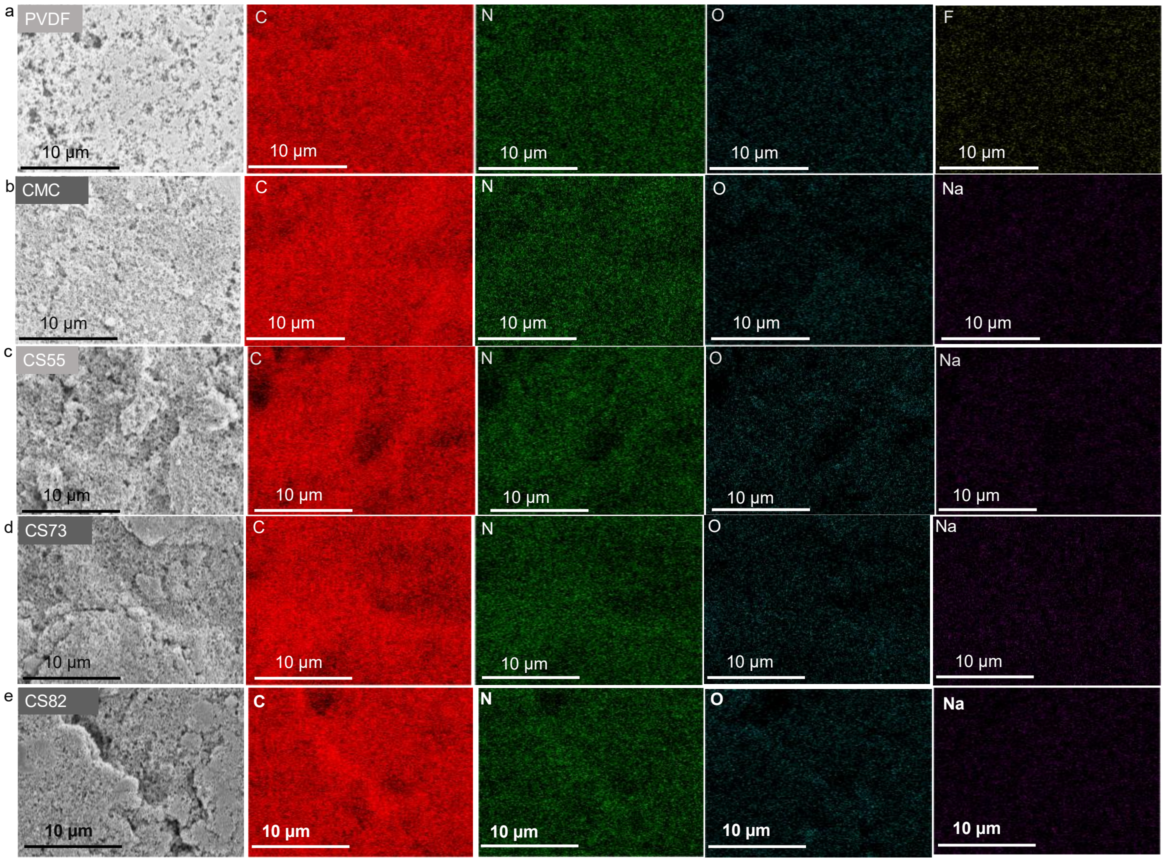

Figure S1: EDS images of the pristine electrodes while using different binders (a)PVdF, (b)CMC, (c) CS 55, (d) CS 73, (e) CS82 electrode. Carbon (C) in red, nitrogen (N) in green, sodium (Na) in magenta, fluorine (F) in yellow, carbon (C) in red, and oxygen (O) in cyan.

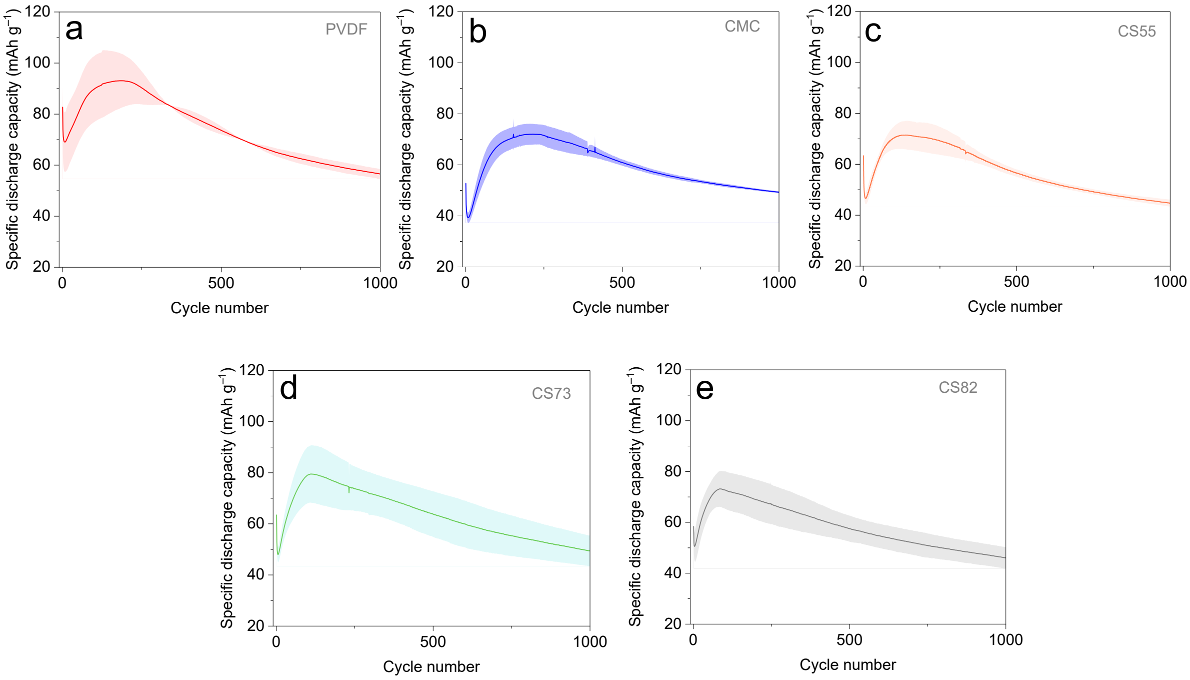

Figure S2: Cycling stability of three measurements and the corresponding standard deviation while (a)PVdF, (b)CMC, (c)CS55, (d)CS73 and (e)CS82 electrodes.

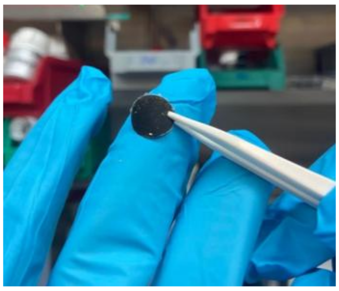

Figure S3: Cycled electrode after 10,000 cycles at 1C rate when CMC binder is used.

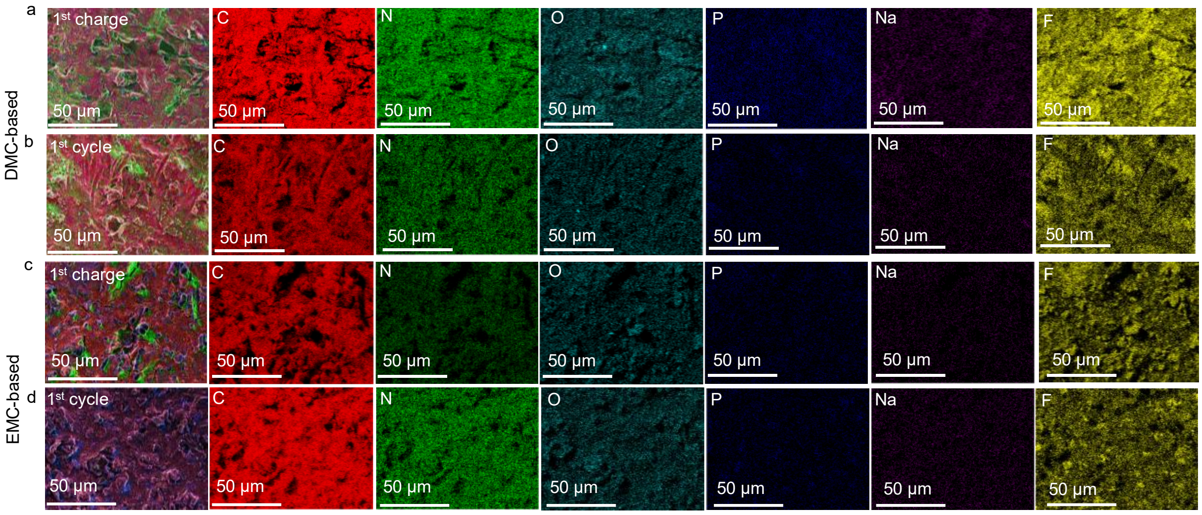

Figure S4: EDS overlap images of the cycled electrodes (1st charge and 1st cycle) while using (a,b) DMC-based and (c,d) EMC-based electrolyte. Carbon (C) in red, Nitrogen (N) in green, oxygen (O) in cyan, phosphorus (P) in blue, sodium (Na) in magenta and fluorine (F) in yellow.

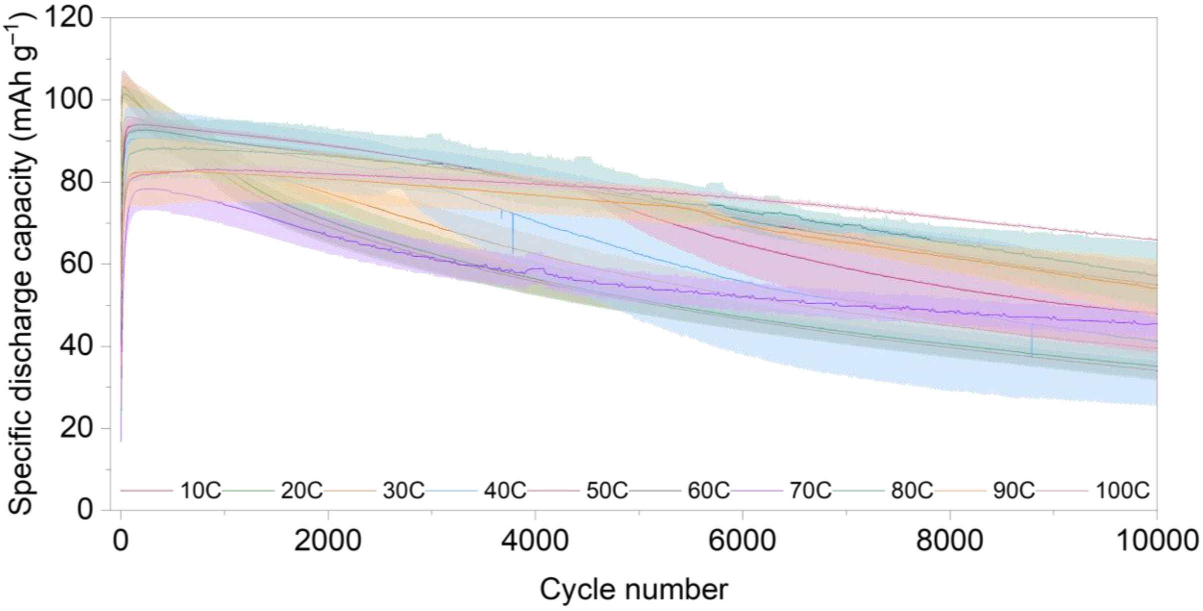

Figure S5: Cyclic stability (Standard deviation plot) of the EMC-based system at higher C-rates.

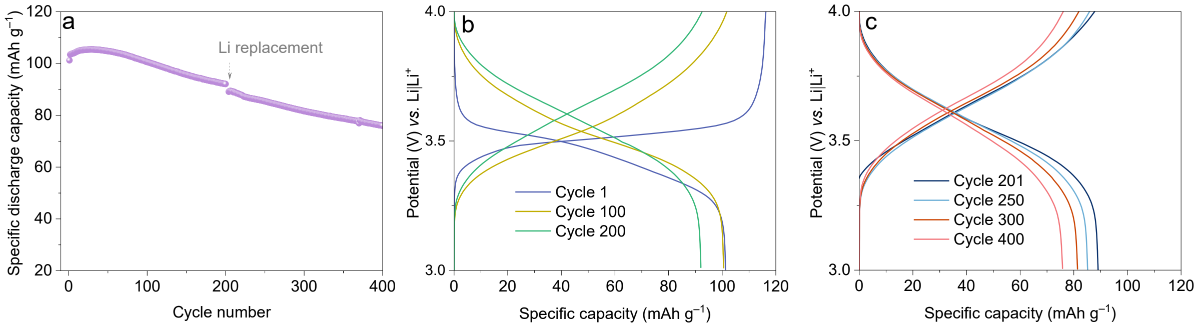

Figure S6: Electrochemical performance of the Li replaced long-term data and the corresponding voltage profiles. (a)Cycling stability plot with lithium replacement after 200 cycles (b) Potential *vs.* capacity profile of the selected cycles from initial to 200 cycles and (c) after Li replacement.

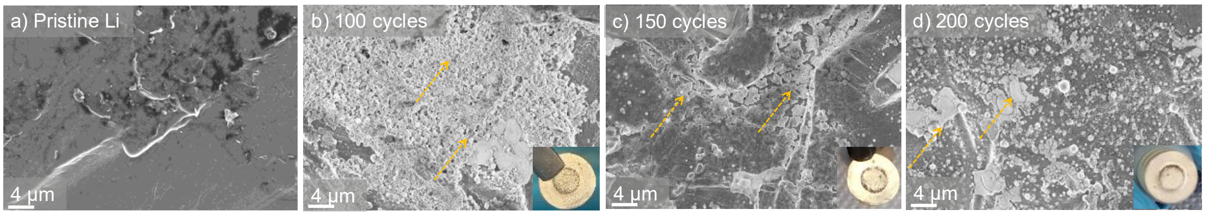

Figure S7: SEM morphology images of (a) pristine lithium and the cycled lithium after (b) 100 cycles (c) 150 cycles and (d) 200 cycles with inset showing the photograph of the corresponding cycled lithium.

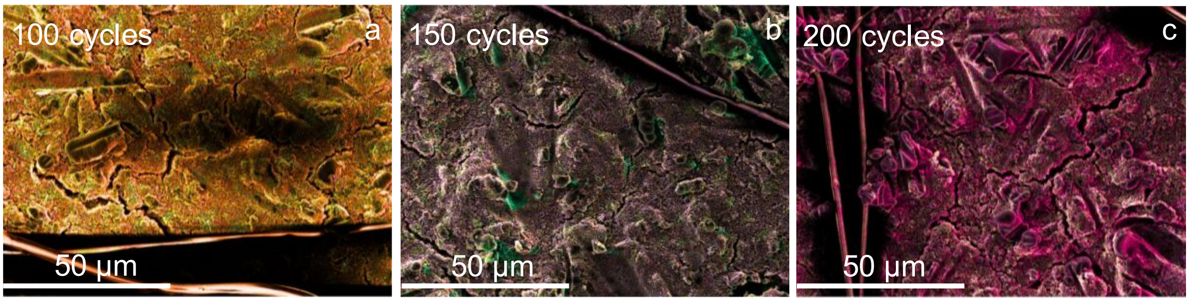

Figure S8: EDS mapping images of the cycled electrodes (a) 100 cycles (b) 150 cycles (c) 200 cycles (carbon - red; nitrogen - green; oxygen - cyan; phosphorous - blue; sodium - magenta; fluorine- yellow)

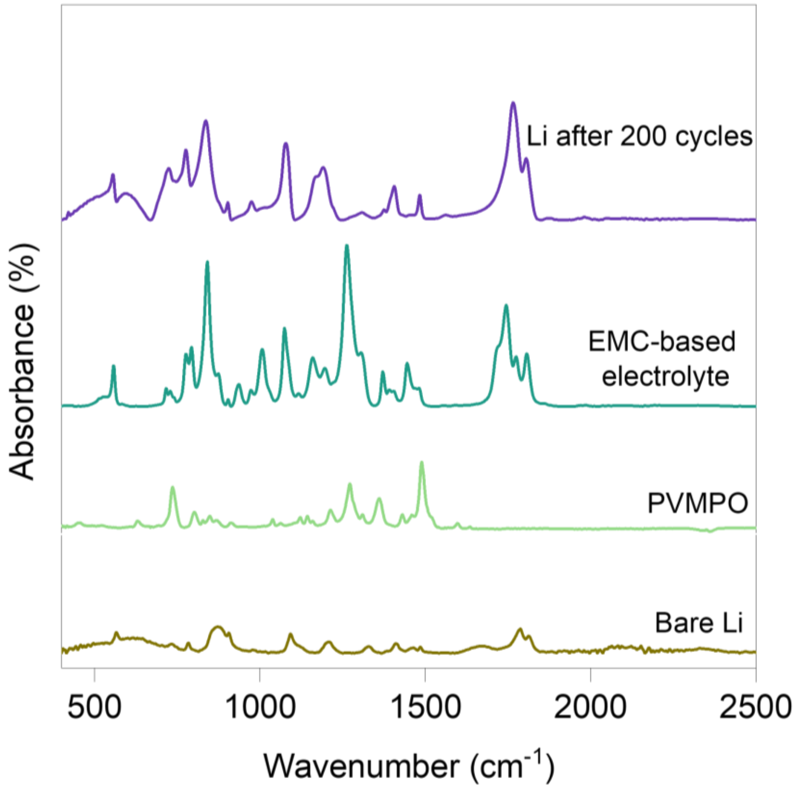

Figure S9: Comparison plot of FT-IR spectrum – Bare Li, pristine PVMPO, EMC-based electrolyte and cycled lithium (after 200 cycles).

Figure S9 shows the comparison FT-IR plots of bare Li, pristine PVMPO, EMC-based electrolyte, and Li after 200 cycles. In the FTIR spectra of cycled Li (after 200 cycles), characteristic C=O

bond stretching vibrations are observed in the region of 1700 to 1800 cm^{-1}. Notably, the C=O stretching band for free ethylene carbonate appear at 1806 cm^{-1}, while the stretching band for ethylene carbonate coordinated with lithium ions is present at 1772 cm^{-1}. Additionally, the infrared spectra exhibit a characteristic P–F bond stretching mode at 838 cm^{-1}. This shows the formation of a carbonate layer over the lithium surface. However, no peaks corresponding to PVMPO can be assigned due to the absence of specific functional group in the polymer.

Table S1: Discharge capacity (DC) and capacity retention (CR) for all the five investigated binder systems at the end of 1000th cycle.

Binder system	1st cycle DC (mAh g^{-1})	200th cycle DC (mAh g^{-1})	500th cycle DC (mAh g^{-1})	1000th cycle DC (mAh g^{-1})	CR 1000th cycle (%)
PVDF	82	93	77	56	68
CMC	53	72	61	49	92
CS11	63	71	56	45	71
CS73	63	76	64	49	78
CS82	58	69	57	46	79

(Capacity retention calculation = Discharge capacity of (nth cycle/1st cycle) *100)

Table S2: The capacity retention (CR) at the end of 100th, 200th and 500th cycles considering 1st cycle specific discharge capacity (DC).

EMC-based	1st cycle DC	100th cycle DC	200th cycle DC	500th cycle DC
Capacity	97	96	89	77
CR (%)	100	99	92	79

Table S3: List of Abbreviations

ACN	Acetonitrile
ATR	Attenuated total reflectance
CCC	Constant current cycling
CMC	carboxymethyl cellulose
CV	Cyclic voltammetry
CVs	Cyclic voltammogram
DMC	Dimethyl carbonate
EC	Ethylene carbonate
EDS	Energy dispersive X-ray spectroscopy
EMC	Ethyl methyl carbonate
FT-IR spectroscopy	Fourier Transform-Infrared spectroscopy
LIB	Lithium ion battery
$LiPF_6$	Lithium hexafluorophosphate
NMP	*N*-methyl-2-pyrrolidone
OCV	Open circuit volatge
PVdF	Poly(vinylidene difluoride)
PVMPO	Poly(3-vinyl-*N*-methylphenoxazine)
Q_{acc}	Accumulated specific discharge capacity
SBR	Styrene-butadiene rubber
SEM	Scanning electron microscopy
TPC	Through-plane conductivity
UV-Vis spectroscopy	Ultraviolet-visible spectroscopy
XRD	X-ray Diffraction
